# *p*-Synephrine Indicates Internal Maturity of *Citrus grandis* (L.) Osbeck cv. Mato Peiyu—Reclaiming Functional Constituents from Nonedible Parts

**DOI:** 10.3390/molecules28104244

**Published:** 2023-05-22

**Authors:** Li-Yun Lin, Chiung Chi Peng, Yi-Ping Huang, Kuan-Chou Chen, Robert Y. Peng

**Affiliations:** 1 Department of Food and Applied Technology, Hungkuang University, 1018, Sec. 6, Taiwan Boulevard, Shalu District, Taichung City 43302, Taiwan; 2 Graduate Institute of Clinical Medicine, College of Medicine, Taipei Medical University, 250 Wu-Shing St., Xin-Yi District, Taipei 110, Taiwan; 3 Department of Urology, Shuang Ho Hospital, Taipei Medical University, 291, Zhong Zheng Rd., Zhonghe, Taipei 23561, Taiwan; 4 Research Institute of Biotechnology, School of Medicine and Nursing, Hungkuang University, 1018, Sec. 6, Taiwan Boulevard, Shalu District, Taichung City 43302, Taiwan

**Keywords:** *Citrus grandis* cv. Mato Peiyu (CGMP), waste reclaim, essential oil, *p*-synephrine, dietary fibers, polyphenols, DPPH, ABTS, antioxidants

## Abstract

The processing of *Citrus grandis* Osbeck cv. Mato Peiyu (CGMP) fruits generates a considerable amount of waste, mainly the flavedo, albedo, and segment membrane; the generated waste yields severe environmental and economic challenges. In this study, we tried to reclaim some functional chemicals from the waste. Our data indicated that the essential oil content in the flavedo was 0.76–1.34%, with the major component being monoterpenes (93.75% in August, declining to 85.56% in November, including mainly limonene (87.08% to 81.12%) and others such as β-myrcene). *p*-Synephrine (mg/100 g dry weight) declined accordingly (flavedo, 10.40 to 2.00; albedo, 1.80 to 0.25; segment membrane, 0.3 in August, 0.2 in September, and none since October). Polyphenols (in μg/g) included gallic acid (70.32–110.25, 99.27–252.89, and 105.78–187.36, respectively); protocatechuic acid (65.32–204.94, 26.35–72.35, and 214.98–302.65, respectively), *p*-coumaric acid (30.63–169.13, 4.32–17.00, and 6.68–34.32, respectively), ferulic acid (12.36–39.36, 1.21–10.25, and 17.07–39.63, respectively), and chlorogenic acid (59.19–199.36, 33.08–108.57, and 65.32–150.14, respectively). Flavonoids (in μg/g) included naringin (flavedo, 89.32–283.19), quercetin (181.05–248.51), nobiletin (259.75–563.7), hesperidin, and diosmin. The phytosterol content (mg/100 g) was 12.50–44.00 in the flavedo. The total dietary fiber in the segment membrane was 57 g/100 g. The antioxidant activity against the DPPH^•^ and ABTS^+•^ free radicals was moderately high. In conclusion, the waste of CGMP fruits is worth reclaiming for essential oil, *p*-synephrine, polyphenolics, and dietary fiber. Notably, *p*-synephrine content (flavedo: <8 mg/100 g dry weight, albedo: <2.0, or segment membrane: <0.4 mg) can serve as a marker of the internal maturation of CGMP fruits.

## 1. Introduction

*Citrus grandis* Osbeck cv. Pejyu Rutaceae (white shaddock) (CGP) is a specialized mutant cultivar of *C*. *grandis* (L.) Osbeck Rutaceae, which was originally introduced to Tainan by D.T. Chen in 1826; it was spread through transplantation by T.S. Chen to southwest Taiwan initially from Mato in 1904 and later to central Taiwan in places such as the Nantou District [1]. It has therefore been named Mato Peiyu (*Citrus grandis* Osbeck cv. Mato Pejyu Rutaceae) (CGMP) since 1927. During the maturation period (late October to November), its average weight reaches 1.00–2.40 kg, making it the world’s largest citrus fruit, with 70–76% pulp and 45% juice (9–13°Bx), 0.5–0.7% acidity, and delicious flavor [2]. In traditional medicine, CGP is used for numerous healing purposes. In some Asian countries, including India, the use of CGP extends beyond fruit consumption: its essential oil and leaves are used for treating skin disorders, headaches, and abdominal pain [3,4,5,6].

Essential oil is the main by-product of citrus fruit processing [7], with the citrus flavedo containing up to 85–99% volatile and 1–15% nonvolatile components [8]. Among nonvolatile components, the *p*-synephrine content in *Citrus* species (Rutaceae) is 0.012–0.099% in unripe fruits and 0.029–0.438% in the leaves [9]. Limonin and nomilin contents in most citrus fruits increase initially and then decrease with fruit growth and maturation, peaking in the young fruit or fruit expansion stage [10]. Volatile constituents include monoterpenes, sesquiterpene hydrocarbons, and their oxygenated derivatives, including aldehydes, ketones, acids, alcohols, and esters [11,12,13,14]. Anmol et al. (2021) reported that the monoterpenes DL-limonene, E-citral, and Z-citral in essential oil are effective antifungal and antiaflatoxigenic agents, whereas sesquiterpenes, aldehydes, ketones, and esters may have antiviral, antioxidant, and bacteriostatic properties [6]. These components are very versatile and are used widely in flavoring, fruit beverages, confectioneries, soft drinks, perfume, soaps, cosmetics, and household products. They also exhibit antimicrobial properties, such as antifungal, antibacterial, antiviral, and antiparasitic activities [8,15]. Given these properties, the global demand for citrus essential oil has markedly increased over the past decades [16].

Fruit and vegetable processing produces some useful by-products, most of which are not exploited commercially, leading to economic and environmental problems [17] due to the high costs of their disposal [18]. Citrus plants are one of the most widely cultivated fruit crops in the world, and their fruit production continues to rise to meet market demand [18]. Consequently, large amounts of waste are generated during citrus juice processing [18], with only approximately 45% of the total fruit weight used and the remaining 55%—peel (flavedo; 27%), albedo and endocarp (26%), and seeds (2%)—being discarded [19].

Dietary fiber (DF) improves intestinal function, facilitates cholesterol reduction, and increases microbial biomass [20]. Soluble DF (SDF) mainly includes β-glucan, pectin, gum, and inulin, and insoluble DF (IDF) includes cellulose, hemicellulose, lignin, cutin, suberin, chitin, chitosan, and resistant starches [20]. The albedo is the white spongy cellulose tissue between the flavedo and the pulp; it is rich in DF [21]. The major properties of DF include solubility, viscosity, water-holding capacity, fermentability, minerals and bile acid–binding ability, oil-binding ability, particle size, and porosity [20]. DF obtained from citrus by-products has a high water-holding capacity, leading to high viscosity, which explains its widespread applications in food [17,22].

Polyphenolics exhibit antioxidant and antihyperlipidemic activities, and flavonoids have antioxidant, antitumor, antibacterial, antiviral, analgesic, and hepatoprotective properties [6].

To overcome the economic and environmental challenges related to the massive waste generated during citrus fruit processing, in this study, we collected the major nonedible parts of citrus fruits that are discarded as waste, such as flavedo, albedo, and segment membrane. From these waste samples, we obtained valuable volatile compounds, such as limonene, and nonvolatile compounds, such as phytosterols, *p*-synephrine, limonin, and DF.

## 2. Results

### 2.1. Weight and Size Variation

**Figure 1:**
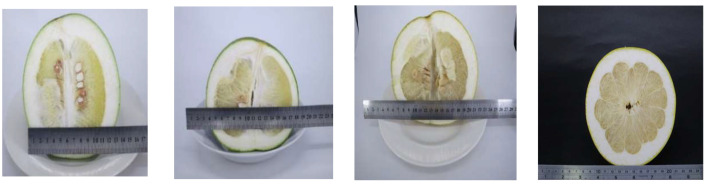
Morphological variation during maturation of Peiyu fruits from August to November.

**Figure 2:**
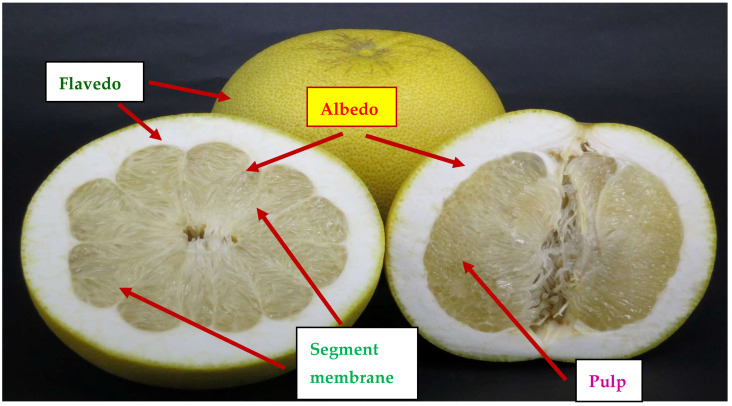
Cross-section of Peiyu fruit showing different parts. The parts used for the experiment were the flavedo, albedo, and segment membrane.

The weight of the CGMP fruit increased from 990 ± 8.32 g in August to 1356 ± 15.98 g in October, and the increase in weight ceased thereafter (Figure 1). Similar trends were noted for the width and length (Table 1). The morphology and color were oblate spherical and yellow, respectively, in October–November from the spherical shape in August (Figure 1). Both the flavedo and albedo became thinner, and the pulp became more spherical, softer, and juicer in October–November (Figure 1 and Figure 2).

### 2.2. Extractability of Essential Oil

**Figure 3:**
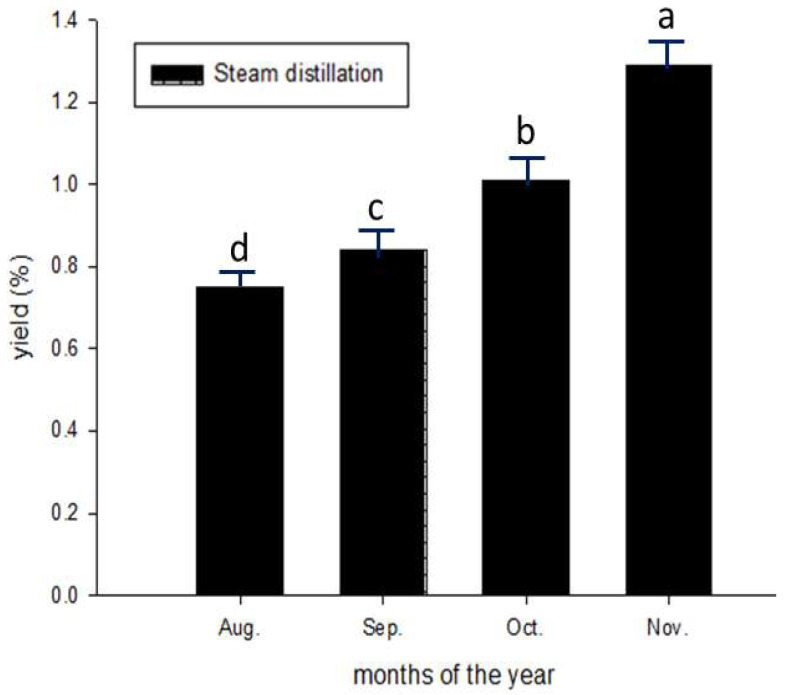
Extractability of essential oils varies during the maturation of Peiyu fruits. Data are expressed as mean ± SD (*n* = 3). Different superscripts in lowercase indicate significance from each other at *p* < 0.05.

The essential oil [24] obtained through water-distilled CGMP oils was transparent. The essential oil yield from the flavedo was 0.76 ± 0.03% in August, increasing to 0.85 ± 0.04% in September, 1.02 ± 0.05% in October, and 1.32 ± 0.05% in November (*p* < 0.05) (Figure 3).

### 2.3. Volatile Components in Essential Oil

The essential oil included 33 volatile compounds: 12 monoterpenes, 9 sesquiterpenes, 3 terpene aldehydes, 6 terpene alcohols, and 3 unclassified oxides. The content may vary depending on the ripening month, but the main monoterpenes were limonene (87.08–81.12%), β-myrcene (2.92–3.17%), β-pinene (0.14–1.37%), β-ocimene (0.06–0.57%), and α-pinene (0.95–1.08%) (Table 2). The major sesquiterpene was germacrene (1.15–1.76%). The major terpene aldehydes included β-citral (0.19–0.84) and α-citral (0.25–0.93) (Table 2). The major terpene alcohols were linalool (0.21–0.30%), cis-geraniol (0.20–0.54%), and α-terpineol (0.14–0.18%). The most commonly found derived oxide was cis-linalool oxide (0.17–0.48%) (Table 2).

### 2.4. Content of p-Synephrine

**Figure 4:**
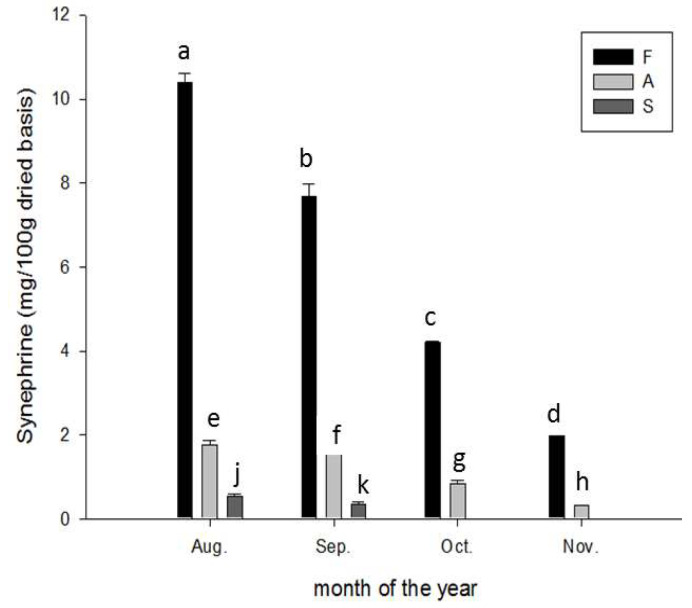
Variation in *p*-synephrine content during maturation of Peiyu fruits. Data are expressed as mean ± SD (*n* = 3). Different superscripts in lowercase indicate significant differences other among groups F (a–d), A (e–h), and S (j, k), respectively, at *p* < 0.05. F: flavedo; A: albedo; S: segment membrane.

The *p*-synephrine content was the highest in the flavedo, followed by the albedo. In August, the *p*-synephrine content (mg/100 g dry weight) reached 10.4 ± 0.4, 1.80 ± 0.1, and 0.04 ± 0.01 in the flavedo, albedo, and segment membrane, respectively (*p* < 0.0001), which decreased sequentially from October (4.15 ± 0.01, 0.08 ± 0.01, and 0.0, respectively) to November (2.00 ± 0.01, 0.25 ± 0.001, and 0.0, respectively, *p* < 0.0001; Figure 4).

### 2.5. Limonin and Nomilin Contents

**Figure 5:**
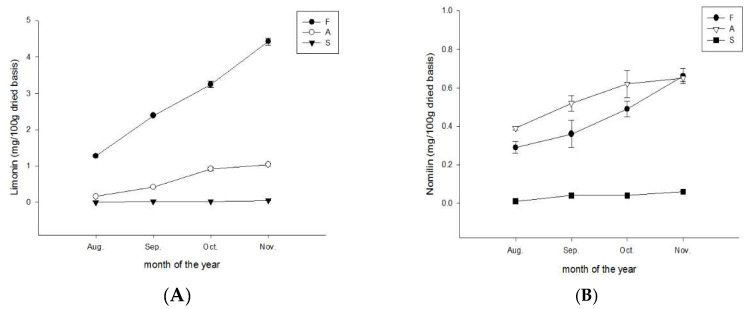
Variation in (**A**) limonin and (**B**) nomilin content during maturation of Peiyu fruits. Data are expressed as mean ± SD (*n* = 3). F: Flavedo, A: Albedo, S: Segment membrane.

Limonin is the bitterest limonoid, followed by nomilin [10]. The limonin and nomolin contents in the flavedo and albedo of CGMP increased depending on the ripening season from August to November. In the flavedo, the limonin content (mg/100 g dry weight) increased from 1.1 ± 0.01 in August to 4.4 ± 0.2 in November (Figure 5A). By contrast, the limonin content in albedo only reached 1.0 ± 0.01 in November and was absent in the segment membrane (Figure 5A). The nomilin content in the flavedo increased from 0.38 ± 0.01 to 0.65 ± 0.03 in November; in the albedo, it increased from 0.29 ± 0.02 in August to 0.66 ± 0.03 in November, whereas the content in the segment membrane did not significantly change (Figure 5B).

### 2.6. Phytosterol Content

**Figure 6:**
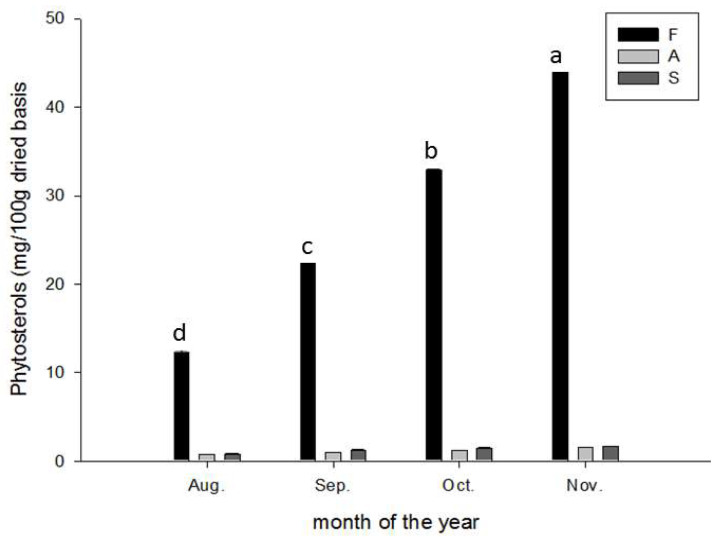
Variation in phytosterol content during maturation of Peiyu fruits. Data are expressed as mean ± SD (*n* = 3). Different superscripts in lowercase indicate significant differences in the flavedo (*p* < 0.05) during the maturation season from August to November. F: flavedo; A: albedo; S: segment membrane.

Phytosterols are natural sterols that are widely found in plants, and they have various physiological functions [25]. The phytosterol content (mg/100 g dry weight) in CGMP increased in a maturity-dependent manner from August (12.50 ± 0.10 mg) through September (23.50 ± 0.20 mg), October (33.50 ± 0.12 mg), and November (44.00 ± 0.20 mg), whereas the content in the albedo and segment membrane were mostly unaffected (Figure 6).

### 2.7. Variation of Total-, Soluble-, and Insoluble DFs

**Figure 7:**
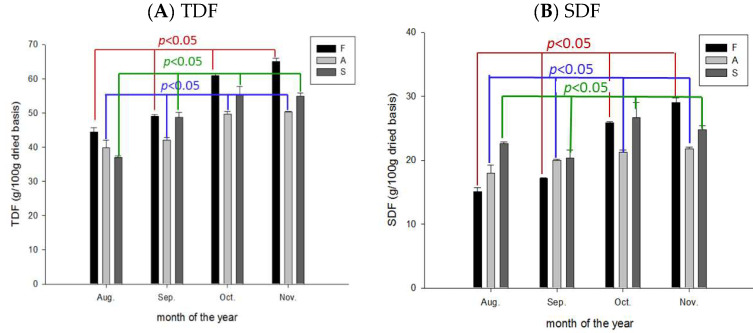

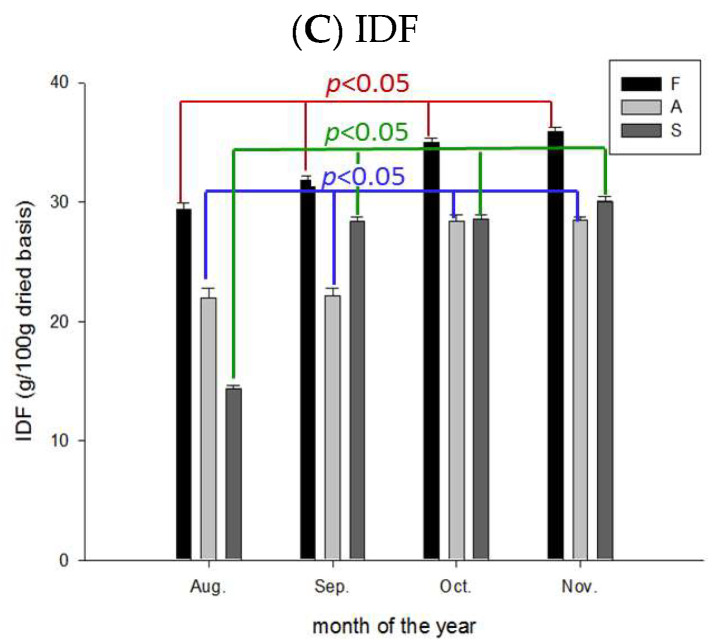
Variation in the content of (**A**) total dietary fiber (TDF), (**B**) soluble dietary fiber (SDF), and (**C**) insoluble dietary fiber (IDF) during maturation of Peiyu fruits. Data are expressed as mean ± SD (*n* = 3). *p* < 0.05 was considered significantly different. F: flavedo (red); A: albedo (blue); and S: segment membrane (green).

The total DF (TDF), SDF, and IDF present in the flavedo, albedo, and segment membranes increased from August to November. The content of TDF in the segment membrane of CHMP was comparable during October and November (57 ± 3 g/100 g vs. 57 ± 1.6 g/100 g dry weight) (Figure 7A). A similar trend was found for SDF (Figure 7B) and IDF (Figure 7C), except for a slight decrease in SDF from October (27.5 ± 3.6 g/100 g dry weight) to November (25.5 ± 1.0 g/100 g) (Figure 7B).

### 2.8. Polyphenolic and Flavonoid Contents

The major polyphenolics found in CGMP were gallic, procatechuic, p-coumaric, ferulic, and chlorogenic acids (Table 3). The contents of all polyphenolic acids in the three parts of the fruit (flavedo, F; albedo, A; and segment membrane, S, except gallic and *p*-coumaric acids) increased from August through November (Table 3), reaching high content in November (in μg/g): procatechuic acid, 204.94 ± 1.52 (F), 72.35 ± 1.65 (A), and 302.65 ± 4.87 (S); ferulic acid, 39.36 ± 2.54 (F), 10.25 ± 1.00 (A), and 39.63 ± 3.26 (S) (Table 3). By contrast, flavonoids were absent in A and S throughout the entire ripening season (Table 4). Naringin, nobiletin, and tangeritin found only in the flavedo decreased with the ripening season. Although hesperidin was found in all parts (F, A, and S), it decreased during ripening (Table 4).

Notably, nobiletin and tangeritin contents were very high in the flavedo (289.36 ± 1.32 μg/g and 563.7 ± 7.36 μg/g, respectively) in early August and decreased somewhat to 80.36 ± 1.65 μg/g and 259.75 ± 2.03 μg/g, respectively, in November (Table 4).

### 2.9. Antioxidant Activity

**Figure 8:**
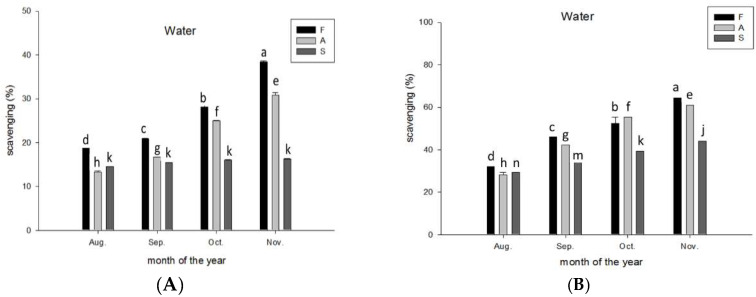
Free radical scavenging activity for (**A**) DPPH^•^ and (**B**) ABTS^+•^ affected by parts, maturation stage of Peiyu fruits, and solvent. Data are expressed as mean ± SD (*n* = 3). Different superscripts in lowercase indicate significant differences in the same part at *p* < 0.05. F: Flavedo (a–d), A: Albedo (e–h), S: Segment membrane (j, k, m, n).

Antioxidant activity of all extracts from the flavedo, albedo, and segment membrane obtained using methanol, ethanol, and water increased with the ripening age of CGMP from August to November (Figure 8), except for the DPPH scavenging activity of the water extract from the segment membrane. DPPH and ABTS scavenging activity were comparable, within a moderate range (20–30%).

## 3. Materials and Methods

### 3.1. Chemicals and Reagents

Methanol, ethanol, *n*-hexane, potassium hydroxide, sodium hydroxide, hydrochloric acid, potassium persulfate, 2,2-azinobis-3-ethylbenzo-thiazoline-6-sulphonic acid (ABTS), and 1,1-diphenyl-2-picrylhydrazyl (DPPH^•^) (all were analytical grade) were supplied by Merck (Billerica, MA, USA). N,O-bis (trimethylsilyl)trifluoroacetamide (*BSTFA*), trimethylchlorosilane (*TMCS*), and 5α-cholestane (purity > 97%) were provided by Sigma-Aldrich (St. Louis, MO, USA). The internal standard 5α-cholestane was prepared in *n*-heptane at −25 °C. Deionized water was obtained using a Millipore purification system with a specific resistance of 18.2 MΩ cm. MES-Tris buffer (pH 8.2) was prepared by dissolving 15.23 g of MES, 3.77 g of Bis-Tris, and 0.725 g of Tris acetate in 2 L of H_2_O, and the buffer was adjusted to pH 8.2.

### 3.2. Source of Peiyu Fruits

CGMP fruits were purchased from a local farm in Nantou District, Taiwan. In the ripening season (August to November in each year) (Figure 1), 40 kg of fresh fruits was selected each month; each batch contained nine fruits (three in each group for triplicate determination). These samples were immediately delivered to our laboratory, where they were rinsed, blow-dried, and peeled. The flavedo, albedo, and segmented membranes were separated for use.

### 3.3. Extraction of Peiyu Essential Oil

To accurately weighed fresh peels (1 kg), 5 L of deionized water was added, blended, and mixed well. The smashed peels were transferred to a 12 L distillation bottle, distilled on a 100 °C steam bath for 4 h, and left to cool to ambient temperature, and the upper layer of essential oils was collected and weighed; extractability was calculated. The essential oils were stored at −20 °C for GC-MS analysis.

### 3.4. Desiccation of the Samples

The flavedo, albedo, and segmented membrane (Figure 2) were separated and blow-dried at 45 °C. The desiccated samples were pulverized to a mesh #100 and then stored in a tightly closed container at room temperature. These samples were used for the analysis of some functional constituents and antioxidant activity.

### 3.5. Analysis of Flavor Compounds

The volatile compounds responsible for the flavor were identified using a GC-MS HP-6890 instrument (Agilent Technologies, Taipei, Taiwan) equipped with a DB-1 capillary column for separation (ℓ × i.d. = 60 m × 0.25 mm, film thickness: 0.25 µm). The flow rate of the carrier gas helium was set at 1.0 mL/min, with a splitting ratio of 50:1. The temperature was programmed as follows: 50 °C initially, an elevation rate of 2 °C/min until 240 °C, and 240 °C was maintained for 20 min. The retention time [3] was used to calculate the retention index (RI) for identifying each volatile compound. The reference compounds used were a mixture of authentic C_5_–C_25_ n-paraffins. The nonisothermal RI was calculated using Equation (1) [26].

(1) $$RI_{x}=100n+100\left(t_{x}-t_{n})/t_{n+1}-t_{n}\right)$$

where *x* denotes the unknown compound, *n* is the carbon number of *n*-alkane, and *t_x_* is the retention time of compound *x*.

The unknown compound was identified by matching using a database. A qualitative approach to identification involved matching with the illustrations in the GC-MSD atlas. The structural deduction of volatile compounds was conducted by referring to the method of Heller and Milne (1978) [24], *Central Institute for Nutrition and Food Research,* TNO (*CIVO*) (1978) [27], National Bureau of Standards computer database, and Brose–Wiley computer database as well as by contrasting with some studies reporting the related mass spectra.

### 3.6. Determination of Phenolics

According to the method of Li et al. (2006), 1 g of desiccated CGMP samples was accurately weighed. Subsequently, 8 mL of methanol (80%) was added, ultrasonicated for 2 h, and centrifuged [25]. To the sediment, 8 mL of methanol (80%) was added, and extraction was repeated. The filtrates were filtered and combined, concentrated to <10 mL at 40 °C under vacuum, and adjusted to a volume of 10 mL by adding 80% methanol; the solution was filtered through 0.22 μm Micropore, 10 μL of which was subjected to high-performance liquid chromatography (HPLC).

A mixture of authentic polyphenols at different concentrations was similarly treated and analyzed using HPLC. The area under the curve was estimated for calculating the polyphenolic content. The HPLC system was equipped with a Hitachi pump L–2130, an RP–18 GP 250 column for separation (ℓ × i.d. = 250 mm × 4.6 mm, Mightysil thickness: 0.32 μm, Kanto Chem), and a Hitachi UV Detector L–2400 monitored at 280 nm. The mobile phase consisted of solution A (10% methanol containing 0.05% formic acid) and solution B (70% methanolic solution containing 0.05% formic acid), and the flow rate was set at 1.0 mL/min. Hitachi D–2000 Elite Chromatography Data Station software was used for analysis.

Polyphenolic determination was conducted using a modified version of the method of Deseva et al. (2020). In brief, 1 g of desiccated CGMP samples was accurately weighed. Subsequently, 8 mL of methanol (80%) was added, ultrasonicated for 2 h, and centrifuged [28]. The supernatant was collected. Extraction was repeated with 8 mL of methanol (80%). The supernatants were combined and filtered, concentrated to <10 mL at 40 °C under vacuum, adjusted to the volume of 10 mL by adding 80% methanol, agitated well, and filtered through 0.22 μm Micropore; 20 μL of this solution was subjected to HPLC.

HPLC was performed using the Hitachi LaChrom Elite HPLC system equipped with a gradient solvent pump coupled to a diode array detector. The separation column used was the Discovery SHC18 column (ℓ × i.d. = 250 mm × 4.6 mm, Supelco thickness: 5 μm) maintained at 30 °C. Gradient elution was performed using a combined mobile phase consisting of solution A (2% [*v*/*v*] acetic acid) and solution B (methanol). Elution was programmed as follows: A:B = 100:0 at 0 min; A:B = 95:5 at 3 min; A:B = 80:20 at 18 min; A:B = 80:20 at 20 min; A:B = 75:24 at 30 min; A:B = 70:30 at 40 min; A:B = 60:40 at 55 min; A:B = 0:100 at 70 min; and A:B = 0:100 at 80 min. The flow rate was set at 1.0 mL/min. Detection was performed at wavelengths of 278 nm for gallic acid and protocatechuic acid; 306 nm for chlorogenic acid, ferulic acid, and *p*-coumaric acid; and 370 nm for rutin and quercetin. Data collection and analysis were conducted using Elite LaChrome software (Tokyo, Japan, Hitachi High-Tech Corporation).

### 3.7. Determination of Flavonoids

Flavonoid analysis was conducted using a method similar to that of phenolics. A mixture of different concentrations of authentic flavonoids was used for calibration. Data collection and analysis were conducted using Elite LaChrome software (Tokyo, Japan, Hitachi High-Tech Corporation).

### 3.8. Assay for p-Synephrine

The *p*-synephrine content was assayed using the method described by Roman et al. (2007) [29], with slight modifications. In brief, 300 mg of desiccated CGMP samples was accurately weighed; 50 mL of 0.1% phosphoric acid solution was added, mixed well, ultrasonicated for 1 h, and left to cool to room temperature. The volume was adjusted to 100 mL with 20 mM boric acid buffer and filtered through 0.45 μm Micropore, and 20 μL of the filtrate was introduced to HPLC. Different concentrations of authentic synephrine solutions were similarly subjected to HPLC. The area under the curve was used to establish the calibration curve, which was used for the calculation of the synephrine content. The HPLC instrument was equipped with a Hitachi pump L-2130, a separation column RP–18GP250 (ℓ × i.d. = 250 mm × 4.6 mm, Mightysil, film thickness: 0.32 μm, Kanto Chem), a Hitachi UV detector L-2400 monitored at UV 224 nm. The mobile phase consisted of solvent A (containing 1.86 g HAS and 20 mM boric acid buffer diluted to 10 mM borate buffer [containing hexane sulfonate]) and solvent B (containing acetonitrile–borate buffer [20:80, *v*/*v*] and 10 mM hexane sulfonate). The flow rate was set at 0.85 mL/min. The results were analyzed using the Hitachi D–2000 Elite Chromatography Data Station.

### 3.9. Determination of Limonin and Nomilin Contents

Limonin and nomilin contents were determined according to the method of Huang et al. (2019) [10]. The desiccated citrus fruit powder (2 g, mesh #100) was defatted with 40 mL of petroleum ether at 20 °C for 12 h, filtered through a vacuum filter (SMB-III; Zhengzhou Great Wall Scientific Industrial, Zhengzhou, China), dried, and ultrasonicated (Elmasonic S100; Elma Schmidvauer GmbH, Bodensee, Germany) at 20 °C for 30 min with 40 mL of acetone. The extract was filtered through 0.5 μm Micropore with the aid of suction and was collected in a 50 mL centrifuge tube. Ultrasonic extraction was repeated twice. The two extracts were combined and centrifuged at 5000× *g* for 10 min at 20 °C. The supernatant was evaporated at 35 °C in a rotary evaporator (Rotavapor R-210; BUCHI Labortechnik AG, Bern, Switzerland) under reduced pressure. The dried residue was re-dissolved in 10 mL of acetonitrile (HPLC grade), the solution was passed through a 0.22 μm PALL syringe filter (JinTeng, Tianjin, China), and 10 μL of the filtrate was subjected to HPLC and monitored at 210 nm; otherwise, the filtrate was stored in the dark until use.

HPLC was conducted using the 1260 HPLC system (Agilent Technologies; Santa Clara, CA, USA) equipped with a separation column C18 HPLC column (ℓ × i.d. = 150 mm × 4.6 mm; film thickness, 5 μm) and maintained at 25 °C. The mobile phase was a mixture of acetonitrile:phosphoric acid buffer (pH 3.5, 0.03 mol/L):methanol = 45:44:11 (*v*/*v*) operated with an isocratic elution model at a flow rate of 1.0 mL/min. Authentic limonin and nomilin were used for calibration.

### 3.10. Determination of Phytosterols

The method of Feng et al. (2006) was followed for the determination of phytosterols, with slight modification [23]. In brief, 5 g of desiccated CGMP samples was accurately weighed, and reflux was extracted with 75 mL of *n*-hexane for 4 h. To the residue, 75 mL of *n*-hexane was added, and extraction was repeated twice. The extracts were combined, filtered, and concentrated under a vacuum to dry. To the dried extract, 50 mL of 6 N HCl ethanolic solution was added, and the mixture was heated at 40 °C for 40 min to facilitate acid hydrolysis. The hydrolysate was evaporated to 5 mL under vacuum and left to cool to room temperature. Subsequently, 2 N KOH-ethanol was added, and the mixture was heated at 60 ± 2 °C for 3 h to accelerate saponification. The saponified solution was neutralized with 2 N HCl ethanolic solution. Thereafter, 75 mL of *n*-hexane was added, and the mixture was transferred to a separatory funnel, agitated vigorously, and left to enable the separation of a clear layer. The *n*-hexane layer was separated, evaporated under vacuum to 2 mL, and decanted into a tube. Next, 100 μL of BSTFA-TMCS (99:1) solution was added, and the mixture was heated at 60 °C for 45 min to facilitate the derivation reaction. The reaction mixture was filtered through a 0.22 μm Micropore filter, left to cool to ambient temperature, and stored in a vial. A 1 μL aliquot was subjected to GC-FID and GC-MS. The internal standard used was 5α-cholestane. GC-FID and GC-MS were conducted using the Agilent GC-MS HP-6890 instrument equipped with an FID detector and a DB-1 capillary column for separation (ℓ × i.d. = 60 mm × 0.25 mm; film thickness, 0.25 μm). The flow rate of the carrier gas helium was set at 1.0 mL/min, with a splitting ratio of 80:1. The operation temperature was programmed as follows: an initial temperature of 200 °C that was maintained for 1 min. The temperature elevation rate was set at 10 °C/min to attain 270 °C for 2 min; then 0.5 °C/min until 280 °C, which was maintained for 1 min; further at 10 °C/min until 290 °C, which was maintained for 10 min.

### 3.11. Analysis of DFs

According to the method described by AOAC (1983), 1 g of desiccated CGMP samples was accurately weighed, and 50 mL 0.05 M MES-Tris buffer (pH 8.2) was added and mixed well [30]. Subsequently, 100 μL thermoresistant a-amylase was added and maintained at 95–100 °C for 30 min with frequent agitation. The reaction mixture was left at ambient temperature, and pH was adjusted to 7.3–7.7 with 0.56 N HCl; 100 μL protease was added, and proteolysis was conducted in a 60 °C water bath for 30 min with frequent agitation. The pH of the reaction mixture was adjusted to 4.0–4.6 with 0.561 N NaOH, and 100 μL amyloglucosidase was added. The mixture was heated in a 60 °C water bath for 30 min with frequent agitation, centrifuged at 8000× *g*, and filtered through the DF crucible with the aid of a suction to separate the water IDF (with the residue retained on the crucible) and the water-soluble dietary fiber (SDF, the filterable portion). The IDF was sequentially rinsed with 10 mL of 78% ethanol and dried at 105 °C in the drier. The weight was measured against a blank and corrected against the ash and the crude protein contents.

### 3.12. Determination of Antioxidant Activity

First, 1 g of desiccated CGMP samples was accurately weighed. Subsequently, 10 mL of 80% methanol, 10 mL of ethanol, and 10 mL of water were added. The mixture was then heated in a 70 °C water bath with constant stirring at 100 rpm for 4 h. The supernatant was collected by decantation, and extraction was repeated twice. The extracts were combined and filtered through Whatman No. 1 filter paper. The filtrate was adjusted to a volume of 10 mL by adding the same solvent, and the mixture was subjected to antioxidant activity tests.

#### 3.12.1. DPPH Free Radical Scavenging Activity

In this study, we followed the method of Lin et al. (2008) [31] for estimating DPPH free radical scavenging activity. Briefly, to 1 mL of the above filtrate, 4 mL of methanol was added and mixed well; then, 1 mL 0.2 mM DPPH methanolic solution was added, mixed well, and left to stand for 30 min. Absorbance was measured at 517 nm. The percent DPPH free radical scavenging activity (% DPPH FRSC) was calculated using Equation (2).

(2) $$\%\;DPPH_{FRSC}=[1-\;(A_{s}/A_{c})]\times 100$$

where *A_s_* is absorbance measured at 517 nm for sample + DPPH in solvent.

*A_c_* denotes the absorbance of the blank DPPH at 517 nm in the solvent.

#### 3.12.2. ABTS^+•^ Free Radical Scavenging Activity

ABTS^+•^ free radical scavenging activity was determined according to the method of Miller and Rice-Evans (1997) [32]. Briefly, 0.25 mL of peroxidase (44 unit/mL), 0.25 mL of ABTS (1000 μM), 0.25 mL of H_2_O_2_ (500 μM), and 1.5 mL of deionized water were mixed and left in the dark for 1 h to facilitate the formation of the bluish-green ABTS cationic free radical (ABTS^+•^). Moreover, 1.5 mL of the precipitate was pipetted, and 0.05 mL of the sample extract was added and mixed for 1 min. Absorbance was measured at 734 nm. The percent ABTS^+•^ free radical scavenging activity (%ABTS^+•^ _FRSC_) was calculated using Equation (3).

(3) $$\%\;\mathrm{ABTS}\;{}_{FRSC}=[1-\;(A_{s}/A_{c})]\times 100$$

where *A_S_* is the absorbance at 734 nm for sample + ABTS^+•^,

*A_c_* is the absorbance of blank ABTS^+•^ at 734 nm.

### 3.13. Statistical Analysis

Triplicate data obtained from the experiments were subjected to statistical analysis with SPSS v10.0 (SPSS, Chicago, IL, USA). One-way analysis of variance was used for within-group comparisons, and Duncan’s multiple range test was used to test the significance of differences between paired means. *p* < 0.05 was considered significantly different.

## 4. Discussion

During ripening, the weight and size of CGMP fruits increased until late October in this study, which is consistent with the finding of Chang et al. (2011a; 2011b) [1,2]. Julhia et al. (2019) suggested that in the harvestability window, the acidity drop and coloration indicate internal maturity, food quality, and harvesting practices [33]. The higher the average fruit weight in a given orchard, the earlier the acidity drop [33]. Because many organic acids, mainly citric acid, serve as respiratory substrates, factors favorable to the enlargement of fruit would thus lead to an earlier acidity drop [33]. The Protected Geographical Indication “Clémentine de Corse.” specifications constrain producers when harvesting clementine; they can harvest clementine under the following conditions: (i) the orange coloration, naturally obtained while on the tree, covers at least 80% of the peel; (ii) a sugar/acid (E/A) ratio between 8 and 17; and (iii) an acid concentration (acidity) between 0.65 g and 1.4 g (expressed as g citric acid per 100 g of juice) [34].

Our extractability for essential oil was comparable to that (0.60–0.79%) reported by Blanco et al. (1995) [35]. Notably, Ou et al. reported much higher yields of essential oils (14.25–16.41%) with cold-press [36] rather than with steam distillation methods [37]. Alternately, Peiyu leaves can also provide essential oils at a yield of 0.13–0.15% [5].

The *p*-synephrine content exhibited a sharp maturity-dependent decrease in the flavedo, albedo, and segment membrane; thus, *p*-synephrine can serve as a distinct molecular marker of the internal maturity of CGMP, which can be assessed together with the chemical signal (the acidity drop) and organoleptic coloration to determine the harvestability window [33], internal biological maturity, food quality, and harvesting practices.

The *p*-synephrine present in CGMP was found mainly in the flavedo, followed by albedo, and its content varied with the maturation stage. In August, the *p*-synephrine content (mg/100 g dry weight) reached 10.4 ± 0.4 (in F), 1.80 ± 0.1 (in A), and 0.04 ± 0.01 (in S), respectively (*p* < 0.0001), and the content decreased sequentially from October (F, 4.15 ± 0.01; A, 0.08 ± 0.01; S = 0.0) to November (F, 2.00 ± 0.01; A, 0.25 ± 0.001, *p* < 0.0001; and S = 0.0) (Figure 4) compared with that in *Citrus* species (ranging from 0.012% to 0.099% in the unripe fruits) [35].

*p*-Synephrine is an adrenergic amine found in many citrus fruits, and it has been used in dietary supplements for weight loss. It may bind to β-3 adrenoreceptors, which are closely associated with thermogenesis, lipolysis, glucose, and cholesterol metabolism, and possibly reduced food intake [38,39,40]. Studies have also demonstrated the high potential of *p*-synephrine for preventing and treating obesity and related metabolic diseases. Synephrine can reduce the activity of C/EBPα and PPARγ and then suppress the adipogenesis of 3T3-L1 cells [41]. The probable mechanism of action involves the activation of the signaling pathway of AMPK-FoxO1 by synephrine, and synephrine also inhibits gluconeogenesis-related enzymes and, subsequently, the production of glucose in hepatocytes [41]. In addition, *p*-synephrine exhibits antidepressant, antiparkinson, anti-inflammatory, and anticancer activities [41].

A low dose (3 mg/kg) of *p*-synephrine is nontoxic [41]. Few studies have analyzed the adverse reactions of dietary supplements containing synephrine [41]. *p*-Synephrine at a dose of 150–2000 mg/kg in mice produced piloerection, gasping, salivation, exophthalmos, and reduction in locomotor activity [9]. Many commercial products containing *p*-synephrine (300 mg/kg), salicin (400 mg/kg), and ephedrine (300 mg/kg) plus caffeine at a 10:4:6:80 *w*/*w* ratio [42,43] were shown to give the expressive number of adverse events [42]. Thus, providing a warning related to its utilization at a high dose is recommended for food safety.

In this study, limonin and nomilin contents increased throughout the maturation period, which is slightly different from the findings of Huang et al. (2019) [10]. Recent studies have demonstrated that the accumulation of limonoids is affected by several factors, including the plant genotype, environment [44,45], tissue, and development stage [46]. Regarding their therapeutic effects, the literature has indicated that limonin and nomilin exhibit chemopreventive and antitumor activities [6].

Notably, CGMP contained abundant maturity-dependent phytosterol. Although the bioavailability of phytosterols is only 0.5–2%, studies have suggested that phytosterols have anti-inflammatory, antibacterial, antifungal, antiulcerative, and antitumoral activities [47,48]. Moreover, phytosterols improve hypercholesterolemia [49,50] by reducing the plasma concentration of total cholesterol, triglycerides, and low-density lipoprotein cholesterol (LDL-C) [25]. In addition, phytosterols can activate the liver X receptor α-CPY7A1, which mediates bile acid excretion and accelerates the transformation and metabolism of cholesterol (Li et al., 2022). The most feasible period for reclaiming such valuable biochemicals from the flavedo (peels) is from October and November.

Moreover, CGMP contains abundant total (TDF), soluble (SDF), and insoluble DFs (IDF).

DF plays an essential role in glucose homeostasis, decreasing total liver lipids, modulating the gut microbiota, and maintaining intestinal health [17,22]. Anmol et al. (2021) reported polysaccharides may also exhibit anti-inflammatory effects [6].

Principally, ABTS^+•^ cationic free radicals can be scavenged by trapping one or two electrons from the antioxidant compounds. Hydrogen transfer plays a major role in DPPH^•^ scavenging. Floegel et al. demonstrated that the antioxidant activity detected using the ABTS^+•^ assay, which was positively associated with the oxygen radical absorbance capacity of total polyphenols and flavonoids in fruits, was stronger than that detected by the DPPH^•^ assay [51], which is in agreement with previous studies (Dudonné et al., 2009; Samaniego Sanchez et al., 2007). Among the food groups, the correlation between antioxidant activities detected using the ABTS^+•^ and DPPH^•^ assays was stronger in fruits and beverages but weaker in vegetables [51].

The high-pigmented and hydrophilic antioxidants were better detected by the ABTS^+•^ assay than by the DPPH^•^ assay [51]. Thus, the higher increase in antioxidant activity of the methanolic and ethanolic extracts from the albedo of CGMP in November indicated the increased production of polyphenolics (Table 3). In agreement with the results of Floegel et al. (2011), the content of gallic acid in all parts decreased from August through November, whereas the contents of procatechuic, *p*-coumaric, ferulic, and chlorogenic acids increased (Table 3); this suggests that the ABTS^+•^ assay is more reliable than the DPPH^•^ assay for detecting antioxidant activity in various foods [51]. Kim et al. (2003) also reported that antioxidant activity measured using the ABTS^+•^ assay was highly correlated with total phenolic content in plums [52], and a weak correlation was found between antioxidant activity and total flavonoids content, which is in agreement with our findings (Table 4).

## 5. Conclusions

The waste generated during CGMP fruit processing contains abundant valuable chemicals that are worth reclaiming. Considering the effect of fruit maturation on these chemicals, the essential oil and phytosterols should be reclaimed from the flavedo in November; *p*-synephrine from the flavedo in August; and DF and polyphenols from the flavedo, albedo, and the segment membrane in October to November. The results can be extended to reclaim the wastes of similar pomelo fruits. Notably, the *p*-synephrine content in the flavedo sharply decreases from August to November, whereas it totally disappears in the segment membrane during the maturation stage from October to November, strongly implicating that *p*-synephrine serves as a reliable internal marker (*p*-synephrine <8 mg/100 g dry weight in the flavedo; <2.0 mg/100 g in the albedo, or <0.4 mg/100 g in the segment membrane) of the physiological maturation of CGMP fruits. We believe that this is the first report of the maturation indicator of *p*-synephrine. Future studies should further treat these samples to expand their application for reclaimed constituents.

## Figures and Tables

**Table 1:** Variation of weight and size of Peiyu fruits during the maturation season from August to November *.

Months	Pejyu		
	Wt (g)	Width [23]	Length [23]
August	990 ± 8.32 ^c^	13.12 ± 1.87 ^a^	13.89 ± 1.83 ^a^
September	1246 ± 9.35 ^b^	14.26 ± 3.98 ^a^	14.02 ± 1.36 ^a^
October	1356 ± 15.98 ^a^	15.00 ± 2.56 ^a^	14.98 ± 3.56 ^a^
November	1356 ± 15.99 ^a^	15.96 ± 5.21 ^a^	15.10 ± 1.32 ^a^

* Data are expressed as mean ± SD from triplicate experiments (*n* = 3). The superscript in lowercase indicates significantly different in the same column (*p* < 0.05).

**Table 2:** Variation of the volatile composition of the essential oil during the maturation season of Pejyu fruits from August to November.

Compounds	RI	CAS-NO	Formula	MW	Composition (%)			
Monoterpene					August	September	October	November
α-thujene	927	002867-5-2	C_10_H_16_	136	0.01	0.02	nd	nd
α-pinene	933	00080-56-8	C_10_H_16_	136	1.09	1.08	1.05	0.95
camphene	948	000079-92-5	C_10_H_16_	136	0.02	0.01	0.01	nd
sabinene	968	003387-41-5	C_10_H_16_	136	0.50	0.44	0.42	0.23
β-pinene	973	000127-91-3	C_10_H_16_	136	1.37	1.08	0.84	0.14
β-myrcene	982	000123-35-3	C_10_H_16_	136	2.99	3.17	3.12	2.92
α-phellandrene	997	000099-83-2	C_10_H_16_	136	0.07	0.02	0.07	0.02
limonene	1042	005989-54-8	C_10_H_16_	136	87.08	85.57	85.42	81.12
β-ocimene	1045	003779-61-1	C_10_H_16_	136	0.46	0.37	0.57	0.06
γ-terpinene	1055	000099-85-4	C_10_H_16_	136	0.03	0.03	0.04	0.01
α-terpinolen	1082	000586-62-9	C_10_H_16_	136	0.03	0.02	0.02	0.02
α-terpinene	1335	000099-86-5	C_10_H_16_	136	0.10	0.08	0.10	0.09
total					93.75	91.88	91.66	85.56
Sesquiterpene								
α-copaene	1376	003856-25-5	C_15_H_24_	204	0.03	0.01	0.01	nd
β-elemene	1387	000515-13-9	C_15_H_24_	204	0.08	0.07	0.07	0.08
γ-cadinene	1417	039029-41-9	C_15_H_24_	204	0.07	0.06	0.05	0.03
β-caryophyllene	1419	000087-44-5	C_15_H_24_	204	0.18	0.11	0.08	0.06
β-cubebene	1443	013744-15-5	C_15_H_24_	204	0.02	0.02	0.02	0.10
α-caryophyllene	1453	006753-98-6	C_15_H_24_	204	0.02	0.01	0.01	0.06
α-amorphene	1472	000483-75-0	C_15_H_24_	204	0.02	0.01	0.02	nd
germacrene-D	1479	023986-74-5	C_15_H_24_	204	1.76	1.50	1.43	1.15
δ-cadinene	1514	000483-76-1	C_15_H_24_	204	0.03	0.02	0.03	0.02
total					2.21	1.81	1.72	1.50
Terpene aldehydes								
β-citronellal	1133	000106-23-0	C_10_H_18_O	154	0.03	0.03	0.04	0.04
β-citral	1218	000106-26-3	C_10_H_16_O	152	0.19	0.22	0.19	0.84
α-citral	1247	000141-27-5	C_10_H_16_O	152	0.25	0.3	0.72	0.93
total					0.47	0.55	1.55	1.81
Terpene alcohols								
linalol	1087	000078-70-6	C_10_H_18_O	154	0.30	0.29	0.28	0.21
4-terpeneol	1166	000562-74-3	C_10_H_18_O	154	0.05	0.05	0.04	0.03
α-terpineol	1177	010482-56-1	C_10_H_18_O	154	0.18	0.16	0.15	0.14
trans-carveol	1202	001197-07-5	C_10_H_16_O	152	0.05	0.07	0.05	0.02
cis-geraniol	1213	000106-25-2	C_10_H_18_O	154	0.20	0.54	0.27	0.26
α-cadinol	1632	000481-34-5	C_25_H_26_O	222	0.01	0.02	0.04	0.01
total					0.79	1.13	0.83	0.67
Other								
cis-linalool oxide	1077	005989-33-3	C_10_H_18_O_2_	170	0.48	0.22	0.21	0.17
limonene oxide	1119	004680-24-4	C_10_H_16_O	152	0.03	0.05	0.04	0.01
trans-limonene oxide	1124	004959-35-7	C_10_H_16_O	152	0.03	0.04	0.03	0.03
total					0.54	0.31	0.28	0.21

RI: Retention indices against the paraffin (C_5_–C_25_) references. nd: not detected.

**Table 3:** Variation of polyphenolic content during the maturation season of Peiyu fruits from August to November ^1^.

^1^ Months	Hydroxybenzoic Acid								
	Gallic Acid (μg/g)				Protocatechuic Acid (μg/g)				
	F	A		S	F		A	S	
August	110.25 ± 3.56 ^a^	252.89 ± 4.23 ^a^		187.36 ± 2.56 ^a^	65.32 ± 2.36 ^d^		26.35 ± 2.36 ^c^	214.98 ± 4.23 ^c^	
September	97.34 ± 0.58 ^b^	175.32 ± 1.25 ^b^		126.36 ± 1.65 ^b^	138.19 ± 165 ^c^		46.84 ± 4.32 ^b^	282.17 ± 3.54 ^b^	
October	90.99 ± 1.36 ^c^	107.65 ± 2.02 ^c^		119.35 ± 0.98 ^c^	173.36 ± 3.25 ^b^		68.64 ± 1.87 ^a^	299.88 ± 1.00 ^a^	
November	70.32 ± 2.35 ^d^	99.27 ± 3.21 ^d^		105.78 ± 1.69 ^d^	204.94 ± 1.52 ^a^		72.35 ± 1.65 ^a^	302.65 ± 4.87 ^a^	
**^1^ Months**	**Hydroxycinnamic Acid**								
	***p-*** **Coumaric Acid (μg/g)**			**Ferulic Acid (μg/g)**			**Chlorogenic Acid (μg/g)**		
	**F**	**A**	**S**	**F**	**A**	**S**	**F**	**A**	**S**
August	169.13 ± 2.89 ^a^	17.00 ± 2.01 ^a^	34.32 ± 0.36 ^a^	12.36 ± 1.32 ^d^	1.21 ± 0.32 ^c^	17.07 ± 2.56 ^d^	59.19 ± 2.54 ^d^	33.081.10 ^d^	65.32 ± 2.03 ^d^
September	72.24 ± 3.02 ^b^	13.48 ± 0.52 ^b^	18.23 ± 3.32 ^b^	20.35 ± 3.32 ^c^	3.32 ± 1.00 ^b^	24.23 ± 1.33 ^c^	98.66 ± 1.23 ^c^	65.51 ± 3.21 ^c^	88.36 ± 0.32 ^c^
October	42.98 ± 4.12 ^c^	7.35 ± 1.25 ^c^	10.36 ± 3.02 ^c^	34.32 ± 1.45 ^b^	9.58 ± 0.48 ^a^	29.01 ± 1.21 ^b^	123.87 ± 3.20 ^b^	84.33 ± 4.23 ^b^	133.11 ± 4.20 ^b^
November	30.63 ± 3.58 ^d^	4.32 ± 0.75 ^d^	6.68 ± 1.02 ^d^	39.36 ± 2.54 ^a^	10.25 ± 1.00 ^a^	39.63 ± 3.26 ^a^	199.36 ± 1.20 ^a^	108.57 ± 1.54 ^a^	150.14 ± 3.02 ^a^

^1^ Data are expressed as mean ± SD (*n* = 3). Different superscripts in lowercase in the same column denote significant differences (*p* < 0.05). F: Flavedo, A: Albedo, S: Segment membrane.

**Table 4:** Variation of flavonoid content during the maturation season of Peiyu fruits from August to November ^1^.

^1^ Months	Glucosides								
	Naringin (μg/g)			Hesperidin (mg/g)			Diosmin (mg/g)		
	F	A	S	F	A	S	F	A	S
August	283.19 ± 4.65 ^a^	nd	nd	6.87 ± 0.56 ^a^	13.34 ± 1.22 ^a^	7.64 ± 0.58 ^a^	5.19 ± 0.54 ^a^	nd	nd
September	213.53 ± 5.32 ^b^	nd	nd	4.10 ± 0.65 ^b^	7.17 ± 0.78 ^b^	5.96 ± 0.98 ^b^	4.66 ± 0.32 ^a^	nd	nd
October	101.76 ± 1.32 ^c^	nd	nd	3.20 ± 0.32 ^bc^	4.51 ± 0.65 ^c^	5.05 ± 0.47 ^b^	1.36 ± 0.85 ^b^	nd	nd
November	89.32 ± 2.32 ^d^	nd	nd	2.73 ± 0.85 ^c^	3.61 ± 0.21 ^c^	4.79 ± 0.00 ^b^	0.85 ± 0.69 ^b^	nd	nd
**^1^ Months**	**Aglycone**			**Polymethoxylated**					
	**Quercetin (μg/g)**			**Nobiletin (μg/g)**			**Tangeritin (μg/g)**		
	**F**	**A**	**S**	**F**	**A**	**S**	**F**	**A**	**S**
August	nd	nd	nd	289.36 ± 1.32 ^a^	nd	nd	563.7 ± 7.63 ^a^	nd	nd
September	nd	nd	nd	156.34 ± 3.20 ^b^	nd	nd	351.18 ± 7.25 ^b^	nd	nd
October	181.05 ± 5.32	nd	nd	116.31 ± 2.01 ^c^	nd	nd	323.39 ± 10.25 ^c^	nd	nd
November	248.51 ± 2.02	nd	nd	80.36 ± 1.65 ^d^	nd	nd	259.75 ± 2.03 ^d^	nd	nd

^1^ Data are expressed as mean ± SD (*n* = 3). Different superscripts in lowercase in the same column denote significant differences (*p* < 0.05). nd: not detected. F: Flavedo, A: Albedo, S: Segment membrane.

## Data Availability

Not applicable.

## Sample Availability

Samples of the compounds are not available from the authors.
